# Understanding the structure and composition of recalcitrant oligosaccharides in hydrolysate using high-throughput biotin-based glycome profiling and mass spectrometry

**DOI:** 10.1038/s41598-022-06530-y

**Published:** 2022-02-15

**Authors:** Saisi Xue, Sivakumar Pattathil, Leonardo da Costa Sousa, Bryan Ubanwa, Bruce Dale, A. Daniel Jones, Venkatesh Balan

**Affiliations:** 1 Departmet of Chemical Engineering and Material Science, Michigan State University, East Lansing, MI 48824 USA; 2 Great Lakes Bioenergy Research Center, Michigan State University, East Lansing, MI 48824 USA; 3 Mascoma LLC (Lallemand Inc.), 67 Etna Road, Lebanon, NH 03766 USA; 4 Department of Biochemistry and Molecular Biology, Michigan State University, East Lansing, MI 48824 USA; 5 Department of Engineering Technology, College of Technology, University of Houston, Sugarland, TX 77479 USA; 6 Complex Carbohydrate Research Center, University of Georgia, 315 Riverbend Rd, Athens, GA 30602 USA

**Keywords:** Biological techniques, Biotechnology, Molecular biology, Plant sciences, Environmental sciences, Biomarkers, Energy science and technology, Engineering

## Abstract

Novel Immunological and Mass Spectrometry Methods for Comprehensive Analysis of Recalcitrant Oligosaccharides in AFEX Pretreated Corn Stover. Lignocellulosic biomass is a sustainable alternative to fossil fuel and is extensively used for developing bio-based technologies to produce products such as food, feed, fuel, and chemicals. The key to these technologies is to develop cost competitive processes to convert complex carbohydrates present in plant cell wall to simple sugars such as glucose, xylose, and arabinose. Since lignocellulosic biomass is highly recalcitrant, it must undergo a combination of thermochemical treatment such as Ammonia Fiber Expansion (AFEX), dilute acid (DA), Ionic Liquid (IL) and biological treatment such as enzyme hydrolysis and microbial fermentation to produce desired products. However, when using commercial fungal enzymes during hydrolysis, only 75–85% of the soluble sugars generated are monomeric sugars, while the remaining 15–25% are soluble recalcitrant oligosaccharides that cannot be easily utilized by microorganisms. Previously, we successfully separated and purified the soluble recalcitrant oligosaccharides using a combination of charcoal and celite-based separation followed by size exclusion chromatography and studies their inhibitory properties on enzymes. We discovered that the oligosaccharides with higher degree of polymerization (DP) containing methylated uronic acid substitutions were more recalcitrant towards commercial enzyme mixtures than lower DP and neutral oligosaccharides. Here, we report the use of several complementary techniques that include glycome profiling using plant biomass glycan specific monoclonal antibodies (mAbs) to characterize sugar linkages in plant cell walls and enzymatic hydrolysate, matrix-assisted laser desorption ionization time-of-flight mass spectrometry (MALDI-TOF-MS) using structurally-informative diagnostic peaks offered by negative ion post-secondary decay spectra, gas chromatography followed by mass spectrometry (GC–MS) to characterize oligosaccharide sugar linkages with and without derivatization. Since oligosaccharides (DP 4–20) are small, it is challenging to mobilize these molecules for mAbs binding and characterization. To overcome this problem, we have applied a new biotin-coupling based oligosaccharide immobilization method that successfully tagged most of the low DP soluble oligosaccharides on to a micro-plate surface followed by specific linkage analysis using mAbs in a high-throughput system. This new approach will help develop more advanced versions of future high throughput glycome profiling methods that can be used to separate and characterize oligosaccharides present in biomarkers for diagnostic applications.

## Introduction

Lignocellulosic biomass comprised of agricultural, forest, herbaceous and woody materials are potential feedstocks for producing biobased products including food, feed, fuels, and chemicals precursors for making higher value products^1^. Carbohydrates such as cellulose and hemicellulose present in plant cell wall are depolymerized to simple sugars using chemical treatment and biological conversions such as enzyme hydrolysis and microbial fermentation^2^. Common pretreatments include Ammonia Fiber Expansion (AFEX), Dilute Acid (DA), Ionic Liquid (IL) and Steam Explosion (SE) that use a combination of chemical and heat to reduce lignocellulosic biomass recalciatrance by opening-up the plant cell wall^3–5^. Enzyme hydrolyses are carried out at high solids loading using commercial enzymes containing Carbohydrate-Active Enzymes (CAZymes) and microbial fermentation using genetically modified yeast or bacteria to produced biobased fuels and chemicals^6^.

The CAZymes in commercial enzymes consist of complex mixtures of enzymes that synergistically break down the complex carbohydrate sugar linkages yielding simple sugars^2,7^. A complex network of aromatic polymer lignin with carbohydrates makes them highly recalcitrant resulting in incomplete sugar conversion accumulating 15–25% of unprouctive oligomeric sugars during enzyme hydrolysis of pretreated biomass as we reported before^8^. This is a universal problem when using different biomass pretreatment methods^9^. Some of the reasons for this bottleneck include enzyme inhibition during hydrolysis or absence or low levels of critical essential enzymes that are necessary to break down sugar linkages in plant biomass^10^. Understanding the sugar composition and structural features such as sugar linkages present in oligosacciardes will help us to improve the sugar conversion during hydrolysis making biobased process cost competitive with petroleum derived products.

Determining the structure of carbohydrates is challenging and are carried out using combination of several techniques such as liquid chromatography (LC)^11,12^, nuclear magnetic resonance specrroscopy (NMR)^13^, capilary electrophoresis (CE)^14–16^, and mass spectrometry (MS)^17,18^. MS technique such as matrix-assisted laser desorption ionization time-of-flight mass spectrometry (MALDI-TOF–MS) provides a versatile method used to identify carbohydrate structures^19^. More recently collision-induced dissociation (CID) tandem MS of sodium ion adduct has been most widely used to determine fingerprints corresponding to linkage positions, anomeric configurations, sequences, and branch locations of oligosaccharides^20,21^.

Glycome profiling is an outstanding tool for intensive identification of carbohydrates linkages^22^. This method uses plant cell wall glycan-directed monoclonal antibodies (mAbs) as probes to understand the complex carbohydrate linkages^23^. More than 250 mAbs are available worldwide that are raised against different linear and branched oligosaccharides with different sugars^24^. Several mAbs has been extensively used to characterize plant cell wall structure, composition, and modifications since there is significant variation depending on the cell types, organs, age, developmental stage, and growth environment of the plants^25,26^. Recently this technique has been used to understand the vesicle populations in plant and animal systems and their respective roles in glycan transport defined by subcellular markers, developmental stages, or environmental stimuli conditions^27^, and determining enzyme activities^28^. Some of the diverse glycans and xylan structures that have been identified include Pectins (P), Xylans (X), Mannans (M), Xylo-Glucans (XylGs), Mixed-Linkage Glucans (MLG), Arabino-Xylan (ArbX), Galactomannan Glycans (GalG), Glucurono-Aarabino-Xylans (GArbX) and Arabino-Galactan (ArbG)^29^.

With all these research efforts, however, only a few studies have focused on the nature of oligosaccharide buildup during High Solids Loading (HSL) hydrolysis in terms of release of oligomeric sugars, changes in the oligomer chain length during the course of hydrolysis, ratios of different DP oligomers and their distribution profiles^30–32^. Meanwhile, even though glycome profiling has been proved to be a useful tool for comprehensive glycan structure analysis, small DP water-soluble oligosaccharides were difficult to estimate using antibody method. Smaller DP oligosaccharides with molecular weight less than 5–10 kDA were not able to bind to the ELISA plates^33,34^, and were washed away before antibodies were added.

We present here, for the first time, by combining a one-step biotinylating procedure for soluble recalcitrant oligosaccharides with glycome profiling using ELISA assay on Avidin-coated plates using mAbs. Complementary oligosaccharide linkage analysis using MALDI-TOF-MS and GC–MS based composition of hydrolyzed sugars using trimethylsilyl (TMS) derivatization corroborate our glycome profiling method. This innovative approach could be developed as a high throughput methodology in the future and used more broadly in biomedical research^35^.

Post-translational modification of enzyme and antibodies such as glycosylation^36^ influence their biological activity. For example, changes in serum protein glycosylation play an important role in inflammatory arthritis and glycosylation changes are used as markers for diagnosis^37^. In literature different glycans are reported to have a high propensity of occurring in various diseases including chronic inflammatory gastrointestinal and liver diseases, viral infection, ovarian, breast and prostate cancer^38–40^. Understanding the glycan structures using glycan directed antibody based ELISA method will provide additional layer of assuredness in disease diagnosis without the need of using sophisticated MS techniques.

## Results and discussion

### Recalcitrant oligosaccharides are a common problem for complete enzymatic hydrolysis of lignocellulose

**Figure 1:**
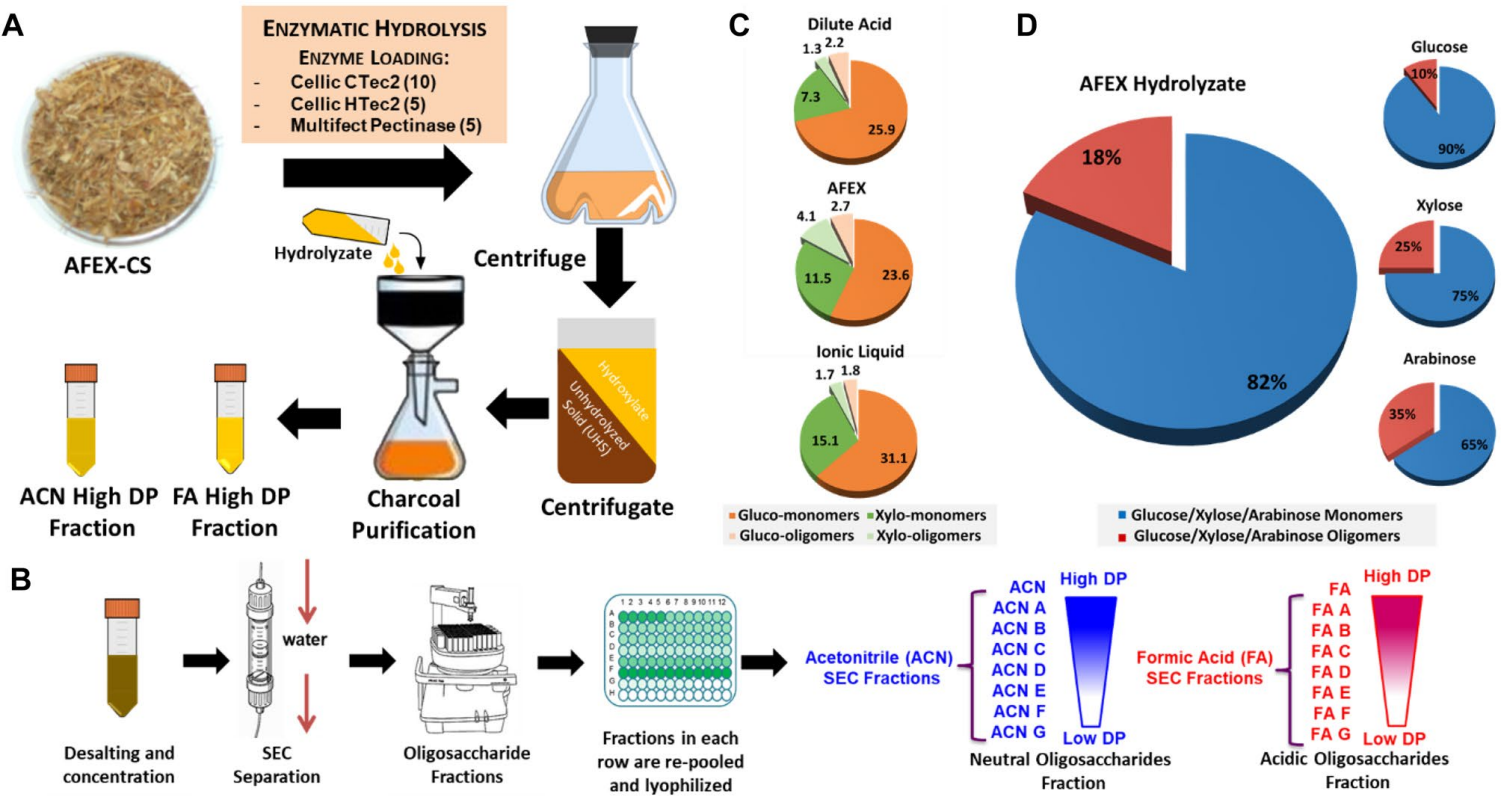
Recalcitrant oligosaccharides remained unhydrolyzed after pretreatment and enzymatic hydrolysis. Here, (**A**) Methodology of oligosaccharides separation where, oligosaccharides were separated from AFEX-pretreated corn stover Hydrolysate (ACSH) by activated charcoal and celite packed bed; (**B**) methodology of oligosaccharides separation. Oligosaccharides were further fractionated by size exclusion chromatography (SEC); (**C**) sugar monomers and oligomers released by different pretreatments (dilute acid: DA, ionic liquid: IL and AFEX). Enzymatic hydrolysis conditions: High solids loading 25% (w/w) (approximately to 8% glucan loading), 96 h hydrolysis, 20 mg/g commercial enzyme loading (Ctec2: Htec2: MP—2:1:1 ratio) and (**D**) Sugar monomers and oligomers of glucose, xylose and arabinose released by AFEX-pretreated corn stover (ACS).

Our previous study found that recalcitrant oligosaccharides remained unhydrolyzed after pretreatment and enzymatic hydrolysis (Fig. 1). In our previously published work, a solid-phase extraction method employing activated charcoal was developed to separate oligosaccharides from AFEX-pretreated corn stover hydrolysate (ACSH)^8^. After the initial extraction and separation, oligosaccharides were further fractionated by Size Exclusion Chromatography (SEC) and collected in the order of molecular weight. Sugar monomers and oligomers released by different pretreatments were analyzed by sugar composition analysis. By comparing the sugar oligomer content resulting from different pretreatment methods, the existence of recalcitrant oligosaccharides is a common problem in biomass conversion to monomeric sugars and can cause at least 10–15% sugar yield loss, and even up to 18% in the case of ACSH. This method was used to further to produce oligosaccharides fractions in large scale. The produced ACSH and its following fractions with varying molecule weight were used as experiment materials in this work for oligosaccharides characterization.

### ELISA-based mAb screening of biotinylated oligosaccharides

**Figure 2:**
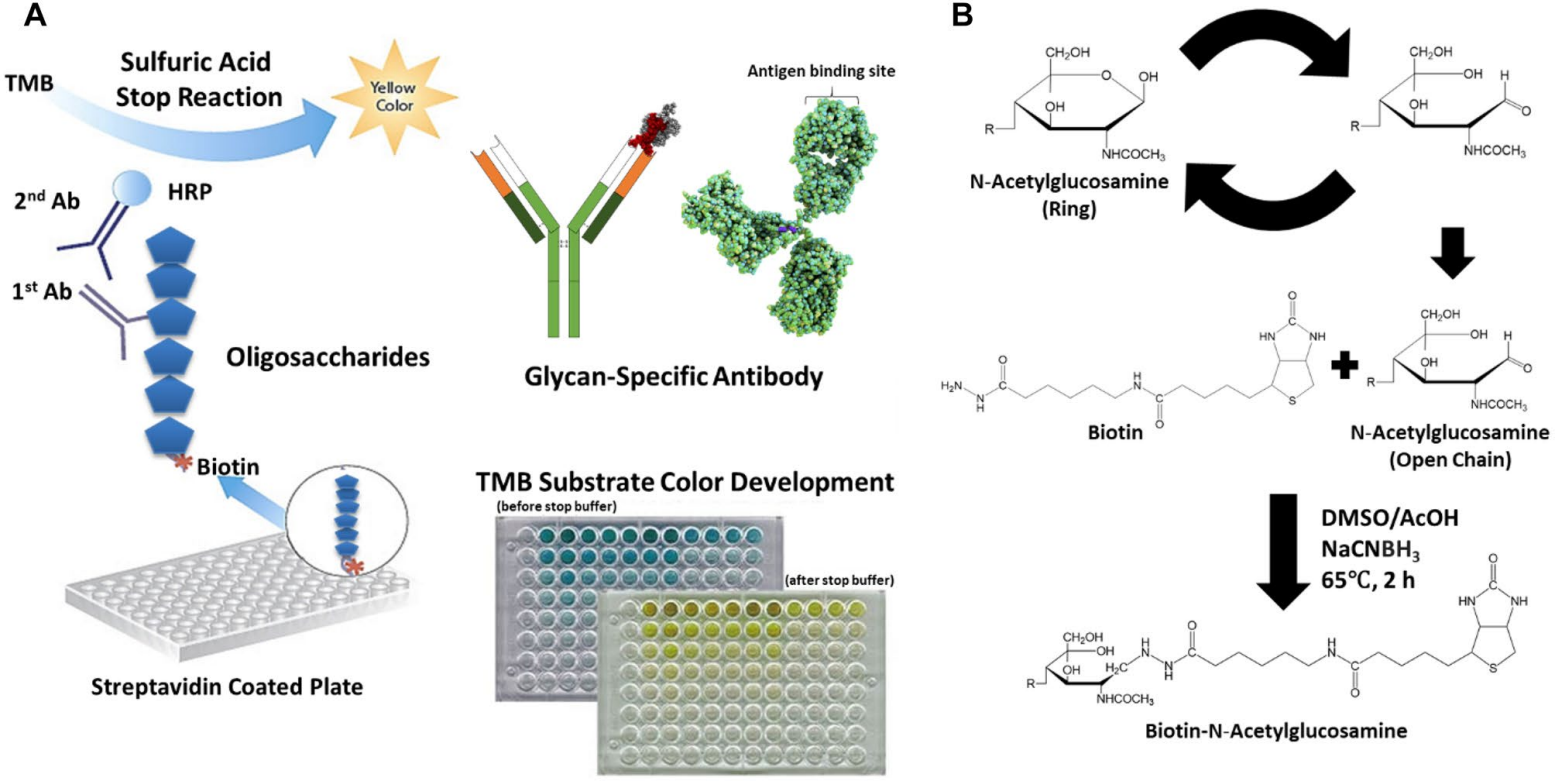
ELISA based monoclonal antibodies screening on biotinylated oligosaccharides. Here, (**A**) combining oligosaccharide biotinylation and subsequent ELISA screening on NeutrAvidin coated plates with glycan-directed mAbs and (**B**) one-step biotinylation procedure showing reaction product.

Glycome profiling has been proven to be a useful tool for comprehensive glycan structure analysis on extracts isolated from solid biomass residues. However, water-soluble sugars were underrepresented using this routine method^41^, as the small molecule oligosaccharides were challenging to be immobilize on the ELISA plates and were washed away before antibodies were added. Thus, a one-step biotinylation technology was applied to enable the coating of soluble recalcitrant oligosaccharides on the Avidin-coated ELISA Plates, enabling the binding with antibody and characterization. The method has been tested using our previously produced ACSH and its fractions based on molecule weight (or degree of polymerization, DP). One-step biotinylation was used to enhance the binding affinity of oligosaccharides (Fig. 2), by adding biotin-LC-hydrazide to the reducing ends of carbohydrates^42^. In solution, the hemi-acetal group at the reducing end reacted with the hydrazide group of biotin-LC-hydrazide to form a hydrazone linkage. In the presence of the reductant NaCNBH_3_, the hydrazone linkage was reduced to become a stable biotinylated end-product. With the sugar reducing end modified, the binding of low-DP oligosaccharides to ELISA plates was then possible and was performed on Avidin-coated plates with glycan-directed mAbs in our study.

The Avidin-coated plates with oligosaccharides binding were then submitted to primary and secondary antibody addition and wash in a light sensitive and time sensitive environment. After the antibody binding was completed, TMB substrate was added to incubate the plates. Finally, the reaction was terminated by sulfuric acid. The incubated plates were analyzed using ELISA reader to determine the binding intensity for each antibody and hence probing against antibody-specific cross-linkages. For experiment details and parameters, please refer to the corresponding “Materials and Methods” section.

### Recalcitrant oligosaccharides epitopes being detected using cell wall glycan-directed mAbs

**Figure 3:**
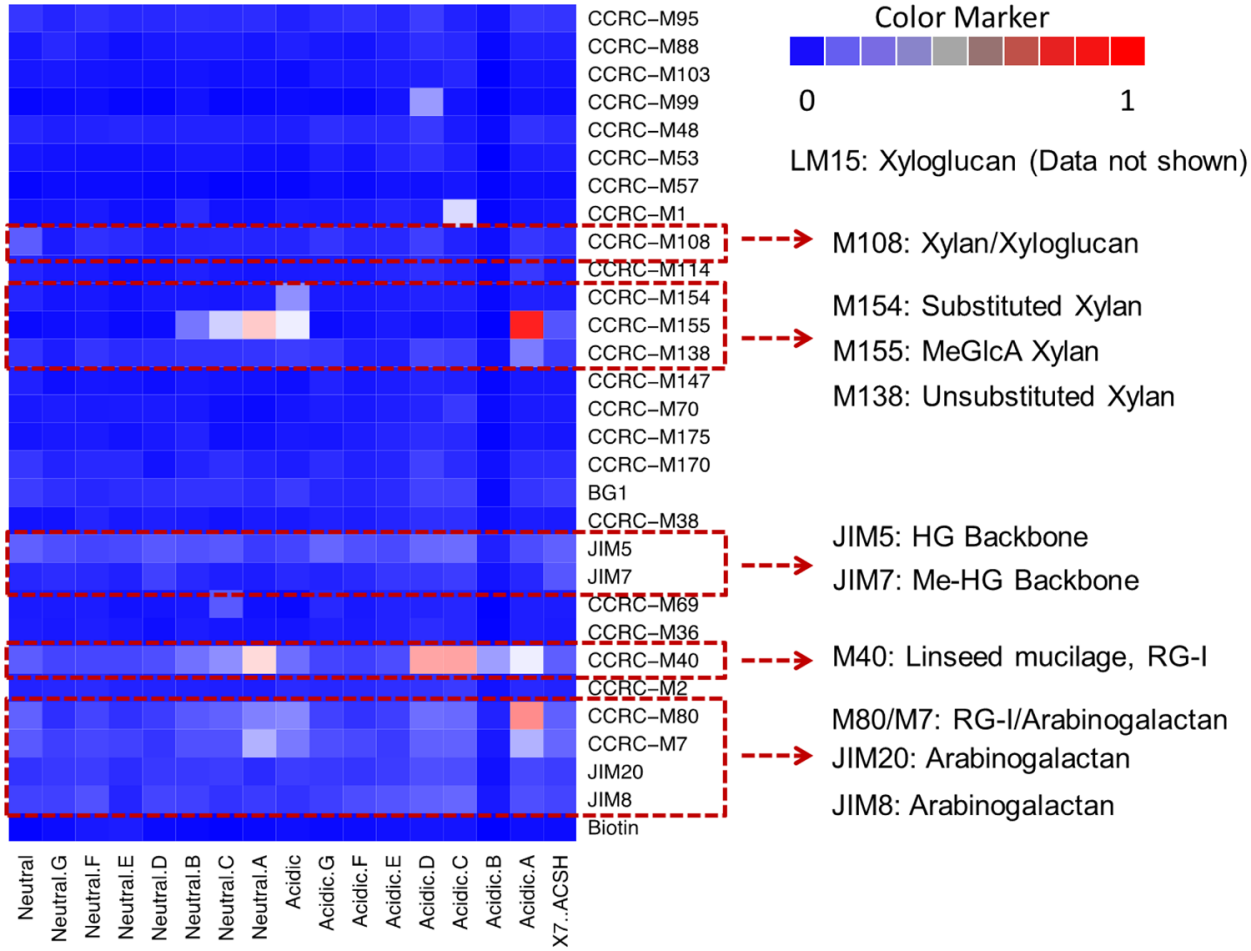
Recalcitrant oligosaccharides epitopes being detected using cell wall glycan-directed monoclonal antibodies. ‘Neutral’ fractions are the ACN fractions and ‘Acidic’ fractions are the FA fractions. Brighter red color in the heat map indicated higher abundance of epitope and brighter blue color indicate blank background. The color value on the scale is based on raw OD values of *N* = 2 preparations. Major epitopes recognized by antibodies are given on the right-hand side.

We proved the value of this newly developed technique in a specific application by characterization of soluble oligosaccharides present in ACSH and in crude and purified oligosaccharides fractions separated from lignocellulosic hydrolysates. As shown in Fig. 3, the most abundant epitopes identified in ACSH using the bionylated glycome profiling method are substituted xylan are usually by uronic acid (U) or methyl uronic acid (MeU) and pectic arabinogalactan. Most of these were also identified in our previous glycome-profiling studies on unhydrolyzed solids (UHS)^43^.

These non-cellulosic structures cannot be digested by the most abundant cellulases and hemicellulases in the tested commercial enzyme cocktails, which have included most of the commonly used commercial enzymes. Their hydrolysis therefore requires novel accessory enzymes. Without the needed non-cellulosic accessory enzymes, these non-cellulosic linkages hinder complete conversion to monomeric sugars, even though their parent sugar polymers were extensively hydrolyzed into shorter fragments and solubilized using commercial enzyme cocktails.

Further examining the distribution of signals and their binding intensity, we observe that the binding epitopes were more prominent in high DP sugar fractions (A, B, C, DP as high as 20+) than in low DP fractions (D, E, F, DP as low as dimer) (Fig. 1). The acidic fractions were more abundant in non-cellulosic epitopes than neutral fractions. These phenomena were consistent with the patterns observed in our previous study^8^, in which high DP and acidic fractions were more resistant to enzyme hydrolysis. Thus, the existence of non-cellulosic glycan epitopes and U and MeU substitutions might contribute significantly to oligosaccharide recalcitrance. It should be noted that binding and detection effectivity for low DP oligosaccharides may have issues particularly if epitopes are dimer or trimer oligosaccharides. This could potentially be verified using commercial oligosaccharides of different lengths which all contain only one epitope that bind to specific mAbs.

The use of structure-specific antibodies has thus identified the certain types of recalcitrant linkages. Based on the types of antibodies being employed, the corresponding linkage pattern and their resulting signal intensity (most and least abundant), new enzymes can be identified and added into enzyme cocktails semi-quantitatively for a more complete sugar conversion. Using ACSH oligosaccharides profiling as an example, we can generate a database of glycan linkages for each biomass material. It should be mentioned here that different affinities of the antibodies should be considered, resulting in certain challenges in comparing between different antibody signals if their affinities are unknown. Also, the glycan linkages comparison may work best between samples for a single antibody. These recalcitrant linkages can then relate to the CAZyme data base, from which we can identify enzymes, select candidates, and screen enzymes to break the bonds, or develop microbial system to express these enzymes for use in a biorefinery^44^.

### Glycosyl-residue composition analyses of recalcitrant oligosaccharides

**Figure 4:**
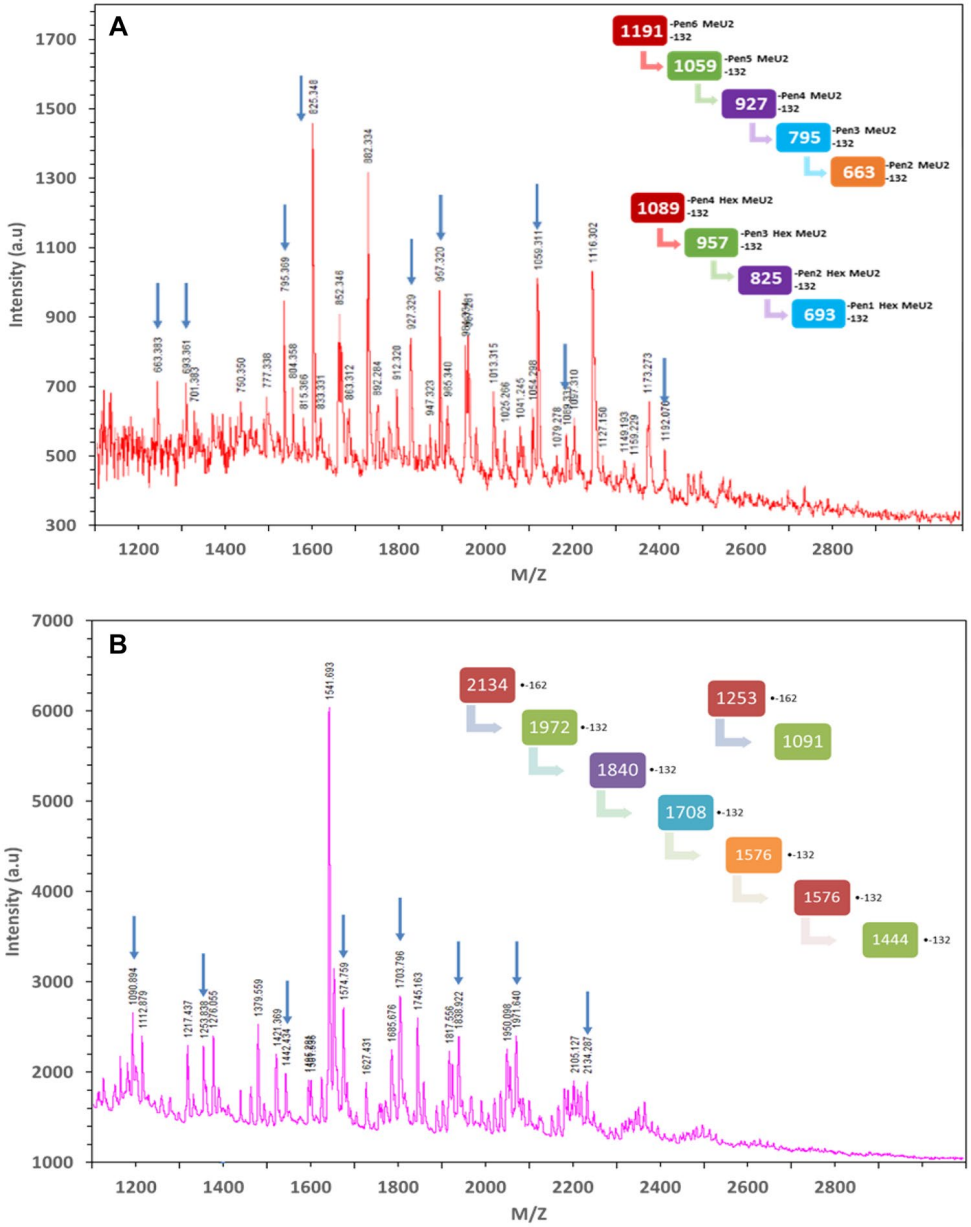
MALDI-MS spectra of soluble recalcitrant oligosaccharides present in ACS. Here, (**A**) ACN-A fraction in low mass range containing glucuronoxylan oligosaccharides with methylated uronic acid substitution (DP 4–8) and (**B**) ACN-B xylan and glucuronoxylan oligosaccharides with methylated uronic acid substitution (DP 8–15).

**Figure 5:**
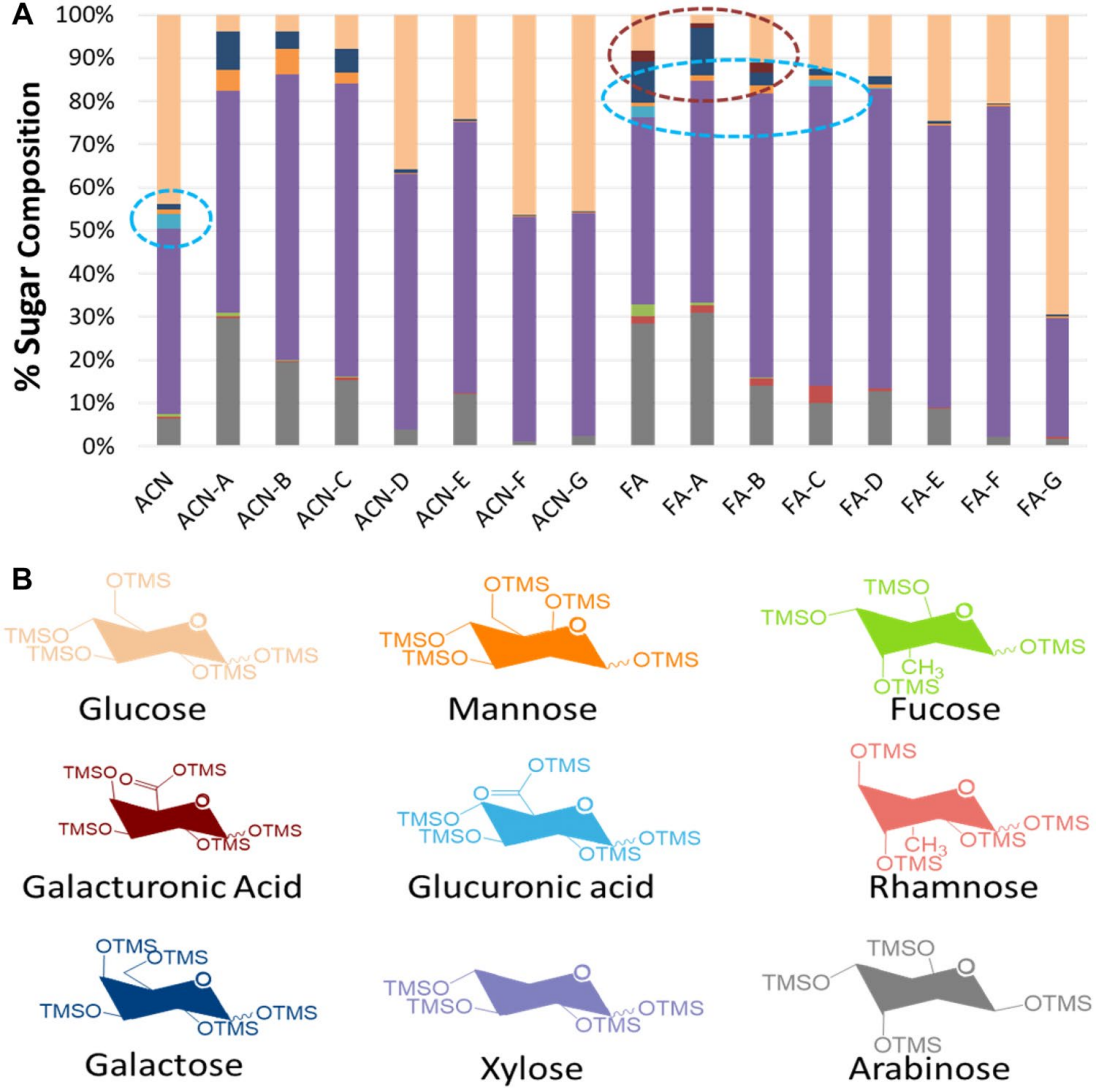
Glycosyl-residue composition analyses of recalcitrant oligosaccharides. Here, (**A**) TMS sugar composition of different oligosaccharides fractions obtained using GC–MS analysis. and (**B**) Structures of different TMS derivatized sugar present in oligosaccharides. *ACN*–acetonitrile fraction containing neutral oligosaccharides and *FA*—Ferulic acid fraction containing acidic oligosaccharides.

To evaluate how immunological methods can complement alternative characterization methods for low molecular weight oligosaccharides present in lignocellulosic hydrolysates, we performed MALDI (Fig. 4, S1-S8) and GC–MS based TMS derivatized sugar analysis (Fig. 5) on the same sets of oligosaccharide fractions. MALDI was used to compare whether the mass distribution of oligosaccharides molecules fit the hypothesized structure. Figure 4 shows the MS of neutral fraction ACN-A and ACN-B. Analyses of ACN-A confirmed Pentose series from DP 4–8 (Fig. 4) up to DP 22 (Fig. S1), the mass of which corresponded with MeU-Xylan oligosaccharides. Analyses of ACN-B confirmed pentose and gluco-xylan series with DP 8–15. In supplementary materials, e.g., Fig. S3, the mass distribution profile of acidic fraction FA-C revealed a (Me)U substituted pentose series with DP 8–15, which was consistent the substituted xylan epitope detected in ELISA based mAb screening.

Another interesting finding comes from the LC–MS analysis of the oligosaccharides fractions as shown in Fig. S9 (Methods can be seen in Electronic Supplementary Materials). Fragments of hexose and –OAc groups are repeatedly observed in ACN-B fraction linkages. This finding not only confirms the fragmentation observed in glycome profiling and MALDI-TOF, but also provides new information on potential carbohydrate derivatives in the pretreated lignocellulosic biomass.

We also performed sugar composition analysis of oligosaccharides fractions using TMS sugar derivatization. Using GC–MS, we determined the composition of both neural (non-derivative) and acidic sugars (GluA and GalA) in oligosaccharide fractions (Fig. 5). Glucoronic acid was found in acidic fraction C and D while galacturonic acid was found in acid fraction A and B, all of which were high-DP acidic sugar fractions. These results not only support our ELISA and MALDI data, but also are consistent with our previous study on oligosaccharides accumulation. Thus, we are confident that an advanced immunological method using oligosaccharides biotinylation and subsequent ELISA screening was adequate to identify soluble recalcitrant oligosaccharides in different biological samples.

### Using commercial oligosaccharides to determine the detection range of glycan-directed mAbs

**Figure 6:**
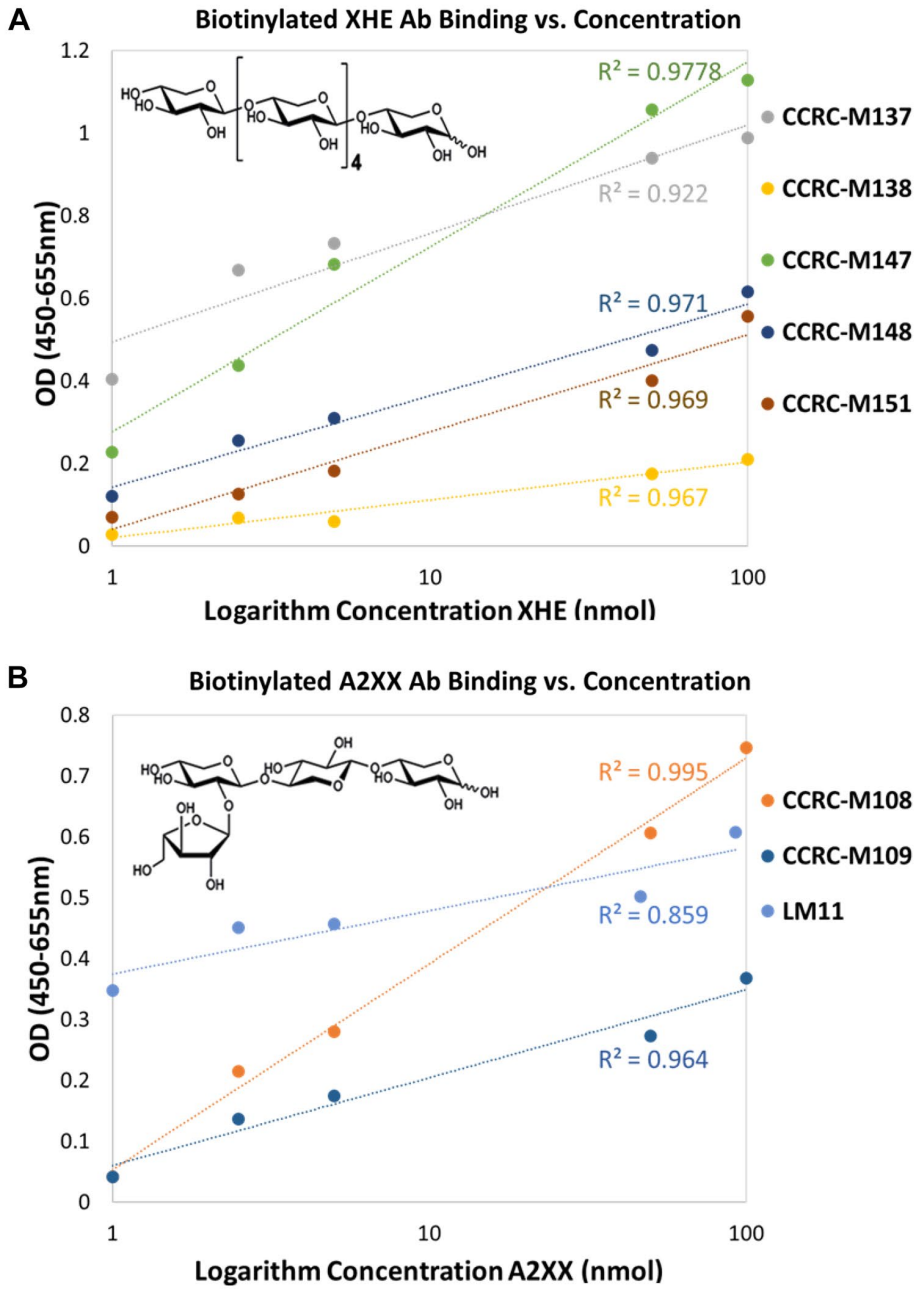
Two commercial oligosaccharides were used to determine the detection range of different glycan-directed monoclonal antibodies. Here, linear correlation with the logarithm concentration of oligosaccharides concentration indicated a Langmuir adsorption model for (**A**) XHE vs. mAbs and (**B**) A2XX vs. mAbs. Respective epitopes indicated the structures in the commercial oligosaccharides that were used as substrates in the assays.

As the ELISA-based mAb screening method has been verified by several different methods, we wanted to further explore the potential of this novel method for quantification. Two commercial oligosaccharides, xylohexaose oligosaccharide (XHE) and 2^3^-α-L-arabinofuranosyl-xylotriose (A2XX), were purchased and tested using the novel cell wall glycan-directed mAbs method. Figure 6 shows the linear correlation between the biotinylated binding signal and the logarithm concentration of oligosaccharides concentration, indicating a possible Langmuir adsorption model. Among the mAbs, CCRC-M137, CCRC-M138, CCRC-M147, CCRC-M148 and CCRC-M151 showed correlation against XHE, and CCRC-M108, CCRC-M109 and LM11 showed correlation against A2XX in the range of 1 nm to 100 nm. Due to the limited availability of antibodies at the time of experiment, limited experiments were performed for each concentration of oligosaccharides. It should be noted here that for the same oligosaccharides as substrate, some antibodies respond very differently, clearly because they bind to slightly different epitopes and may have very different binding affinity. When applying the novel mAbs method to a real sample, the mechanism and consequence of what the epitopes recognize exactly will be far more complicated.

## Conclusion

Use of glycan-directed monoclonal antibodies (glycome profiling or ELISA-based mAb screening) is a powerful tool for in-depth characterization of most major cell wall glycans constituting plant biomass. Classical glycome profiling, however, only facilitates characterization of larger cell wall-glycans, because most oligosaccharides are not efficiently immobilized on ELISA plates. In this study, AFEX pretreated corn stover was subjected to enzymatic hydrolysis at high solid-loading. Glycome profiling was employed to determine the composition of recalcitant cell wall carbohydrates in the hydrolysate. Analysis of smaller oligosaccharides in the hydrolysate using mAbs, however, was underpresented and requiring additional tools to efficiently immobilize oligosaccharides on ELISA plates.

We report here a novel efficient method to immobilize oligosaccharides for mAb screening by combining oligosaccharide biotinylation with the subsequent ELISA screening on NeutrAvidin™ coated plates. The immobilized biotinylated oligosaccharides showed sufficient affinity for the antibodies enabling rapid and efficient detection of recalcitrant oligosaccharides. Mass spectrometry based composition analysis of these recalcitrant oligosaccharides confirmed the results from this novel immuno-screening method. These studies thus demonstrate that combining oligosaccharide biotinylation and ELISA screening with glycan-directed mAbs is useful in identifying the cross-linkages in oligosaccharides and might be widely applied to other biochemical studies characterizing oligosaccharide structures.

This biotin-based glycome profiling method is the first report to enable probing recalcitrant carbohydrate linkages of soluble oligomeric sugars in plant biomass. This has help to understand why certain portion of biomass is highly recalcitrant while producing biofuels. This method fills an important gap in glycome profiling technology and extends its application to a broader spectrum of substrates other than plant oligosaccharides. In the future, we can use robot to do biotynylation and analyze the samples using ELISA in a high-throughput fashion using the methods we developed.

## Materials and methods

### Biomass

Corn Stover (CS) grown from Pioneer hybrid seed variety 33A14 was harvested in 2010 from Kramer farm in Wray, CO. Permission was obtained from the land owner to use this biomass for research. The samples were stored dry < 6% moisture in zip-lock bags in room temperature. The study complies with local and national guidelines. Composition analysis was performed using the NREL protocol^45^. The composition was found to have 31.4% glucan, 18.7% xylan, 3.3% arabinan, 1.2% galactan, 2.2% acetyl, 14.3% lignin, 1.7% protein, and 13.4% ash.

### Chemicals and enzymes

Cellic® CTec2 (138 mg protein/mL, batch number VCNI 0001) is a complex blend of cellulase ezymes, β-glucosidase and Cellic® HTec2 (157 mg protein/mL, batch number VHN00001) is a complex blend of hemicellulase enzymes were generously provided by Novozymes (Franklinton, NC, USA). Multifect Pectinase® (72 mg protein/mL) is a comlex mixture of pectine degrading enzymes was a gift from DuPont Industrial Biosciences (Palo Alto, CA, USA). The protein concentrations of the enzymes were determined by estimating the protein (and subtracting the nonprotein nitrogen contribution) using the Kjeldahl nitrogen analysis method (AOAC Method 2001.11 by Dairy One Cooperative Inc., Ithaca, NY, USA). Celite 545 was purchased from EMD Millipore (Billerica, MA). Activated charcoal (DARCO, 100 mesh particle), Avicel (PH-101), beechwood xylan and all other chemicals were purchased from Sigma-Aldrich (St. Louis, MO).

### AFEX pretreatment

AFEX pretreatment was performed at the GLBRC (Biomass Conversion Research Laboratory, MSU, Lansing, MI, USA). Pretreatmet was carried out at 140 °C for 15 min. residence time at 60% (wt/wt) moisture with 1:1 anhydrous ammonia to biomass loading in a bench-top stainless steel batch reac-tor (Parr Instruments Company)^46^. It took 30 min. for the reactor to reach 140 °C and the ammonia was rapidly released, which quickly brought the biomass to room temperature. The AFEX-pretreated corn stover (ACS) composition was similar to untreated corn stover (UT-CS).

### High solids loading enzymatic hydrolysis

High solids loading 25% (w/w) (approximately to 8% glucan loading) ACSH was prepared as starting material for the large-scale production of oligosaccharides. Enzymatic hydrolysis of ACS was performed using a commercial enzymes mixture including Cellic® Ctec2 10 mg protein/g glucan (in pretreated biomass), Htec2 (Novozymes, Franklinton, NC), 5 mg protein/g glucan and Multifect Pectinase (Genencor Inc, USA), 5 mg protein/g glucan. Enzymatic hydrolysis was carried out in a 5L bioreactor with 3L working volume at pH 4.8, 50 °C, and 250 rpm. After 96 h hydrolysis, the hydrolysate was harvested by centrifugation at 6,000 rpm for 30 min and then 14,000 rpm for 30 min to remove unhydrolyzed solids. Hydrolysate was then sterile filtered through a 0.22 mm filter cup. The filtered hydrolysate was stored at 4 °C in a sterile bottle prior to charcoal fractionation.

### Biomass analysis

Extractive-based compositional analyses of the biomass samples were performed according to the NREL Laboratory Analytical Procedures: Preparation of samples for compositional analysis (NREL/TP-510-42620) and determination of structural carbohydrates and lignin in biomass (NREL/TP-510-42618)^47^.

### Oligosaccharide analysis

Oligomeric sugar analysis was conducted on the hydrolysate liquid streams using an autoclave-based acid hydrolysis method at a 2 mL scale. Hydrolysate samples were mixed with 69.7 μL of 72% sulfuric acid in 10 mL screw-cap culture tubes and incubated in a 121 °C bench-top hot plate for 1 h, cooled on ice and filtered into High Performance Liquid Chromatography (HPLC) vials. The concentration of oligomeric sugar was determined by subtracting the monomeric sugar concentration of the non-hydrolyzed samples from the total sugar concentration of the acid hydrolyzed samples.

### Analytical method

Glucose, xylose and arabinose concentrations in acid hydrolyzed biomass were analyzed using a Shimadzu HPLC system equipped with a Bio-Rad Aminex HPX-87H column equipped with automatic sampler, column heater, isocratic pump, and refractive index detector. The column was maintained at 50 °C and eluted with 5 mM H_2_SO_4_ in water at 0.6 mL/min. flowrate.

### Liquid and solid composition analysis

The hydrolysate supernatants were diluted and analyzed for monomeric and oligomeric sugar contents. Monomeric sugars produced after enzyme hydrolysis were analyzed using an HPLC equipped with a Bio-Rad (Hercules, CA) Aminex HPX-87P column and de-ashing guard column. Column temperature was held at 80 °C and water was used as the mobile phase flowing at 0.6 mL/min. Oligomeric sugars were determined via dilute acid hydrolysis at 121 °C according to the method reported in the literature^41,48,49^.

### Glycome profiling

Glycome profiling of untreated, AFEX-pretreated and all unhydrolyzed biomass residues (involving preparation of sequential cell wall extracts and their mAb screenings) were carried out using the procedures previously described^27,43,50,51^. To conduct glycome profiling, alcohol insoluble residue of plant cell wall materials were prepared from biomass residues and were subjected to sequential extractions with increasingly harsh reagents such as ammonium oxalate (50 mM), sodium carbonate (50 mM with sodium borodeutiride at 0.5% w/v), KOH (1 M and 4 M, both containing sodium borodeutiride at 1% w/v) and acidic chlorite as described previously^52,53^. The extracts were then subjected to ELISA against a comprehensive suite of cell wall glycan-directed mAbs^50^ and the mAb binding responses were represented as heat maps. Plant cell wall glycan-directed mAbs were purchased from laboratory stocks (CCRC, JIM and MAC series).

### ELISA-based mAb screening of biotinylated oligosaccharides

1. **One-step biotinylation of oligosaccharides.** The coupling of carbohydrates to biotin-LC-hydrazide was carried using the following procedure. Biotin-LC-hydrazide (4.6 mg/12 μmol) was dissolved in dimethyl sulfoxide (DMSO, 70 μl) by vigorous mixing and heating at 65 °C for 1 min. Glacial acetic acid (30 μl) was added, and the mixture was poured onto sodium cyanoborohydride (6.4 mg/100 μmol), which dissolved completely after heating at 65 °C for approximately 1 min. Then 5 to 8 μl of the reaction mixture was added to the dried oligosaccharides (1–100 nmol) to obtain a tenfold or greater molar excess of label over reducing ends. The reaction was carried out at 65 °C for 2 h, after which the samples were purified immediately. In labeling experiments without reduction, sodium cyanoborohydride was omitted and the samples were allowed to react at 65 °C for 2.5 h.
2. **ELISA of biotinylated oligosaccharides samples coating and wash.** A 25 μL of biotinylated sample (100 μL each concentrated sample diluted in 5 mL 0.1 M Tris Buffered Saline (TBS) separately) were added to respective wells on Avidin-coated plates. The control wells were coated with 50 μl biotin at 10 μg/mL in 0.1 M TBS. DI water was used as coat for blank readings. The plates were incubated at room temperature for 2 h in the dark. The plates was washed 3 times with 0.1% skimmed milk in 0.1 M TBS using plate washing program #11 for Grenier flat 3A.
3. **Primary antibody addition and wash.** A 40 μl primary antibody was added to respective wells. The microplates were incated at room temperature for 1 h in the dark. The plates were then washed 3 times with 0.1% milk in 0.1 M TBS using plate washing program #11 for Grenier flat 3A.
4. **Secondary antibody addition and wash**. A 50 μl Mouse/Rat secondary antibody was aded to respective wells (dilute secondary antibody in 1:5000 proportion using 0.1% milk in 0.1 M TBS). The microplates was incubated at room temperature for 1 h in the dark. Then the microplates was washed 5 times using 0.1% milk in 0.1 M TBS using plate washing program #12 for Grenier flat 5A.
5. **Substrate Addition.** A 50 μl of 3,3′,5,5′-Tetramethylbenzidine (TMB) was added to the substrate substrate (Prepare TMB substrate by adding 2 drops of buffer, 3 drops of TMB, 2 drops of hydrogen peroxide in 15 mL DI Water and vortex before use). The microplates was incubated at room temperature for 30 min. in the dark.
6. **Termination step and reading the plate.** A 50 μl of 1 N sulfuric acid was added to each well and the absorbance was recorded using ELISA reader between 450 and 655 nm.

### TMS sugar composition analysis

A 1 mg/mL solutions of these analytes was prepared in deionized water: arabinose, rhamnose, fucose, xylose, galacturonic acid (GalA), glucuronic acid (GlcA), mannose, glucose, galactose, N-acetylmannosamine (manNAc), N-acetyl-glucosamine (glcNAc), N-acetyl-galactosamine (galNAc), *myo*-inositol (internal standard). Two standards were prepared by addding the 1 mg/mL sugar solutions as Table 1. The samples were frozen at −80 °C and lyophilize until all water is removed (usually it takes about 12–18 h).

**Table 1:** Standard TMS mix preparation.

Std TMS 1	Std TMS 2
50 μL arabinose	50 μL rhamnose
50 μL fucose	50 μL xylose
50 μL galA	50 μL glcA
50 μL glucose	50 μL mannose
50 μL glcNAc	50 μL galactose
50 μLmanNAc	50 μL galNAc
20 μL inositol	20 μL inositol

### TMS sample preparation

A 100–500 μg of sample was added into a screw cap tube on analytical balance. Record the amount added. It is best to have the samples dissolved in solvent at certain concentration, added into the tube as liquid aliquots. A 20 μL of 1 mg/mL myo-inositol was used as internal standard into each sample tube. The amount of internal standard added to sample must be the same as that added into the standard tube.

### Preparation of 1 M methanolic HCI (to perform up to 20 tubes)

A 8 mL of anhydrous methanol was added into a screw cap test tube. A 4 mL of 3 N methanolic HCl was then added and cap was placed and Vortex. No water used in this process.

### Hydrolysis

A 500 μL 1 M methanolic HCl was added into oligosaccharideds sample and standard TMS tubes. The samples was incubate overnight (168 h) at 80 °C in heat block. Methanolysis product was dried at room temperature using the drying manifold. A 200 μL MeOH was added and dried again. The process was repeated for two times. A 200 μL methanol, 100 μL pyridine, and 100 μL acetic anhydride was added to the sample and mixed well. The sample was incubated at room temperature for 30 min. and dried. A 200 μL methanol was added and dried again.

### Silylation

A 200 μL Tri-Sil was added and the capped tubes were heated for 20 min. at 80 °C and then cooled to room temperature. The samples were further dried using drying manifold to a volume of ~ 50 μL. It is important to note that we did not allow the sample to completely dry.

### Gas chromatography (GC) samples preparation and injection

A 2 mL hexane was added and mix well by vortexing. Pasteur pipette tip (5–8 mm) was pack a bit of glass wool by inserting the glass wool from the top of 5–3/4 -inch pipette. The samples were centrifuged at 3000×g for 2 min. to precipitate any insoluble residue. The samples were dried until it reached 100–150 μL. About 1 µl volume was injected into the GC–MS with initial temp 80 °C and initial time 2.0 min (Table 2).

**Table 2:** GC oven temperature program.

No	Rate	Final temp	Hold time
1	20 °C/min	140 °C	2 min
2	2 °C/min	200 °C	0 min
3	30 °C/min	250 °C	5 min

### Calculation

If running standards for first time, perform TMS derivatization of each sugar separately and run them individually to procure the profiles of retention times, spectra, and distributions of peaks for each monosaccharide. These profiles will be used to identify the monosaccharide in standards mixture and samples. From the GC–MS, obtain the peak area and retention time of each sugar in standards and samples. Assign the corresponding sugar identities to the unknown peaks in samples according to the standards retention time. Because each sugar can have as many as 4 peaks, in total all the area from peaks corresponding to 1 sugar.

Calculate the detector response factor (RF) value of each sugar in the standard mixture against internal standard (in this case, inositol).

$$\mathrm{RF\,of\,glucose}= \frac{\mathrm{total\,peak\,areas\,of\,glucose\,or\,inositol\,in \,standard}}{\mathrm{glucose\,or\,inositol\,weight\,in\,standard}}$$

Calculate the mass of each sugar in the sample in mg using the RF value for corresponding sugar.

$$\mathrm{glucose\,amount }(\mathrm{ug})= \frac{\mathrm{sample\,glucose\,peak\,area}*\mathrm{inositol\,mass\,in\,sample}}{\mathrm{RF\,of\,glucose}*\mathrm{samples\,inositol\,peak\,area\,Inositol}}$$

Calculate the mol% of each sugar Calculate the number of moles of each sugar in sample.

$$ {\text{No}}.\;{\text{of}}\;{\text{umoles}} = {\text{ }}\frac{{{\text{mass}}\;{\text{of}}\;{\text{sugar}}\;({\text{in}}\;{\text{ug}})}}{{{\text{MW}}\;{\text{of}}\;{\text{sugar}}\;({\text{in}}\frac{{{\text{ug}}}}{{{\text{umol}}}})}} $$

Calculate the total *µ*moles of all the sugars present in the samples. Calculate the mol% of each sugar per total moles of all sugars.

$$\mathrm{\%\,sugar\,composition}= \frac{\mathrm{sugar\,amount }(\mathrm{ug})}{\mathrm{sample\,amount }(\mathrm{ug})}$$

## Supplementary Information

Supplementary Information.
