# Supplementary material for: Understanding the structure and composition of recalcitrant oligosaccharides in hydrolysate using high-throughput biotin-based glycome profiling and mass spectrometry
Source: Sci Rep. 2022 Feb 15;12:2521. doi: 10.1038/s41598-022-06530-y (PMC8847591; doi:10.1038/s41598-022-06530-y)
Supplement: Supplementary file 1 — Supplementary Information. [file 41598_2022_6530_MOESM1_ESM.docx]

**Supplementary Figures:**


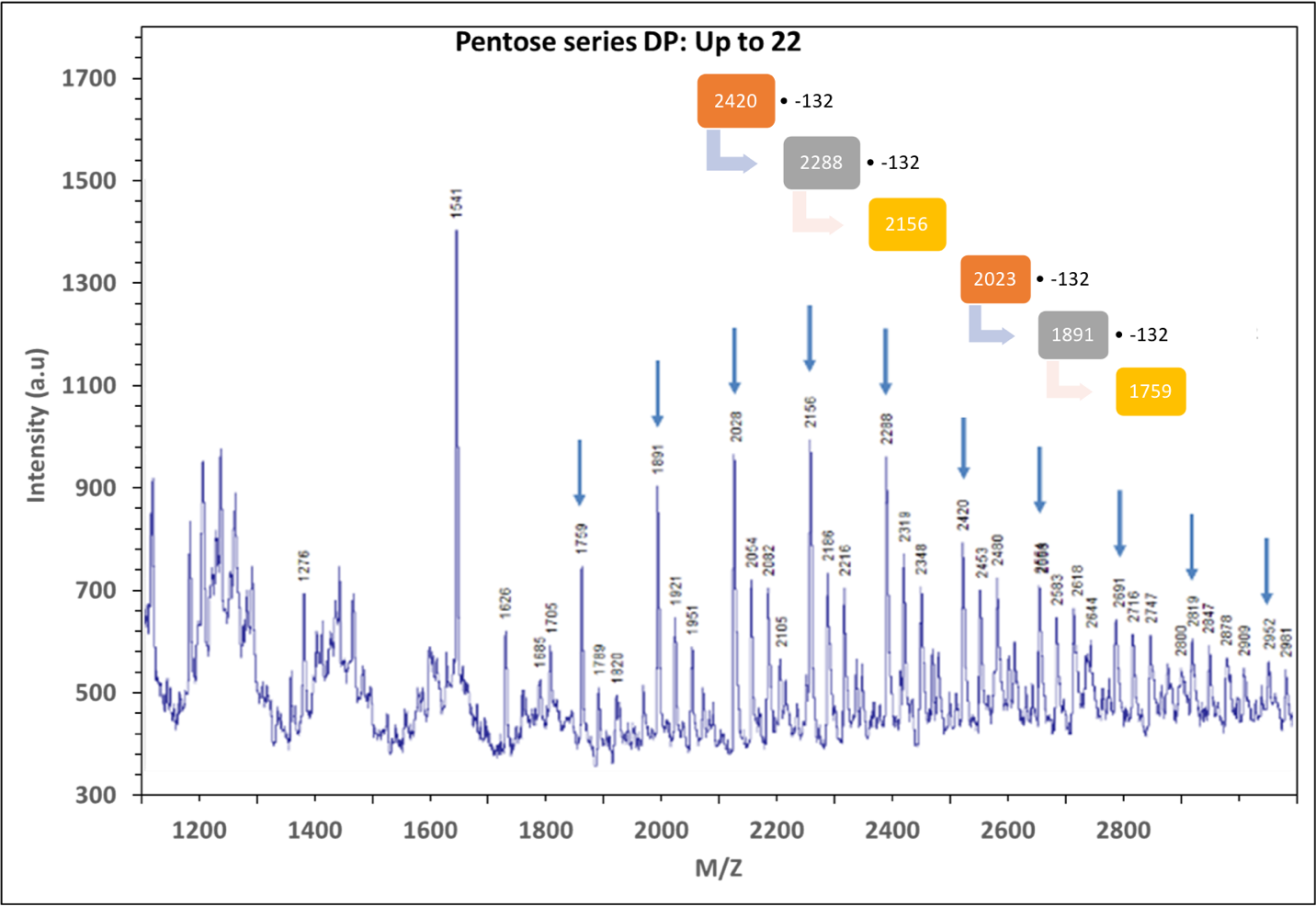


**Figure S1.** MALDI Analyses of ACN-A (high –DP) confirmed Pentose series up to 22.

**
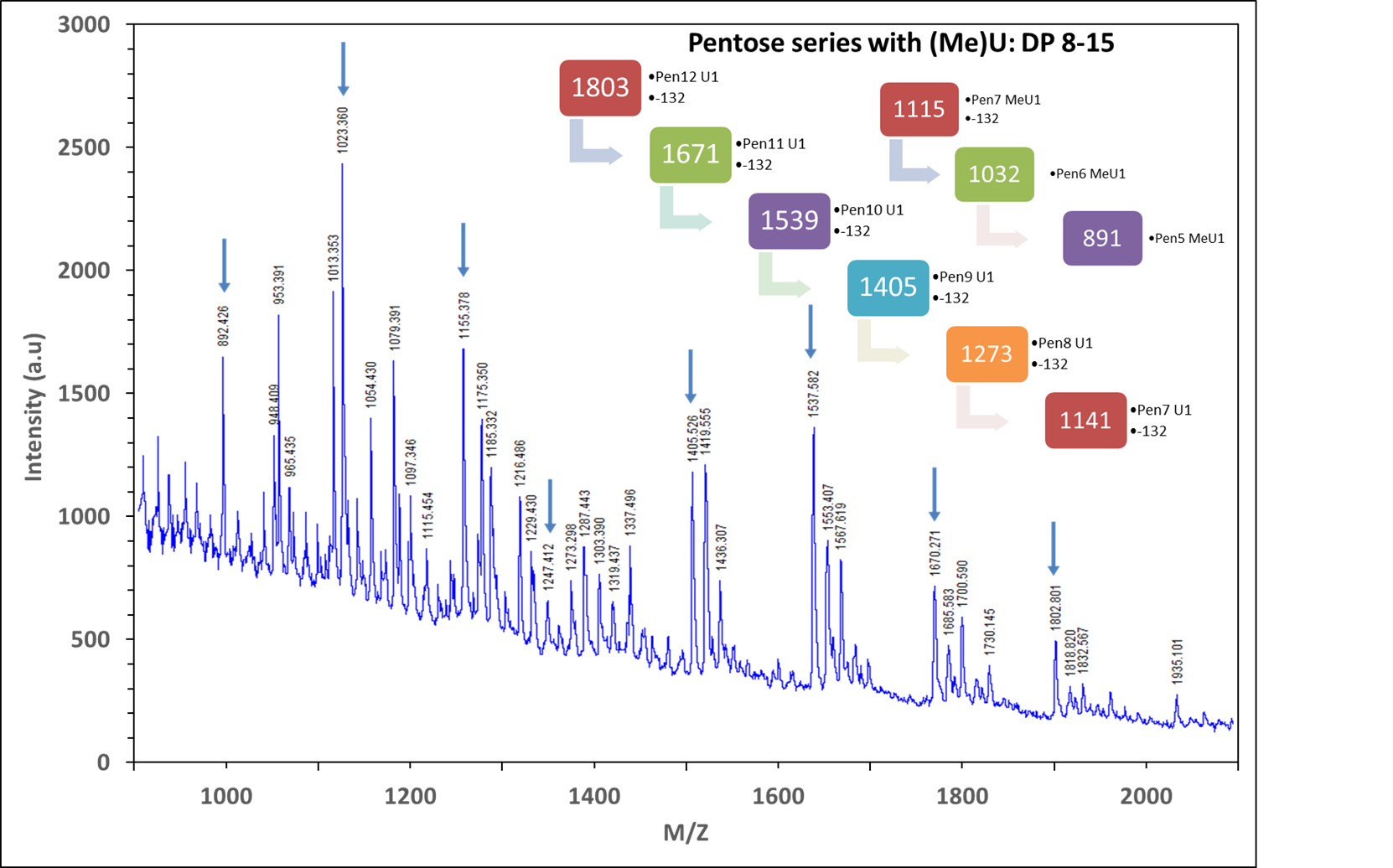
**

**Figure S2.** MALDI Analyses of ACN-C confirmed MeU Xylan series: DP 8-15.


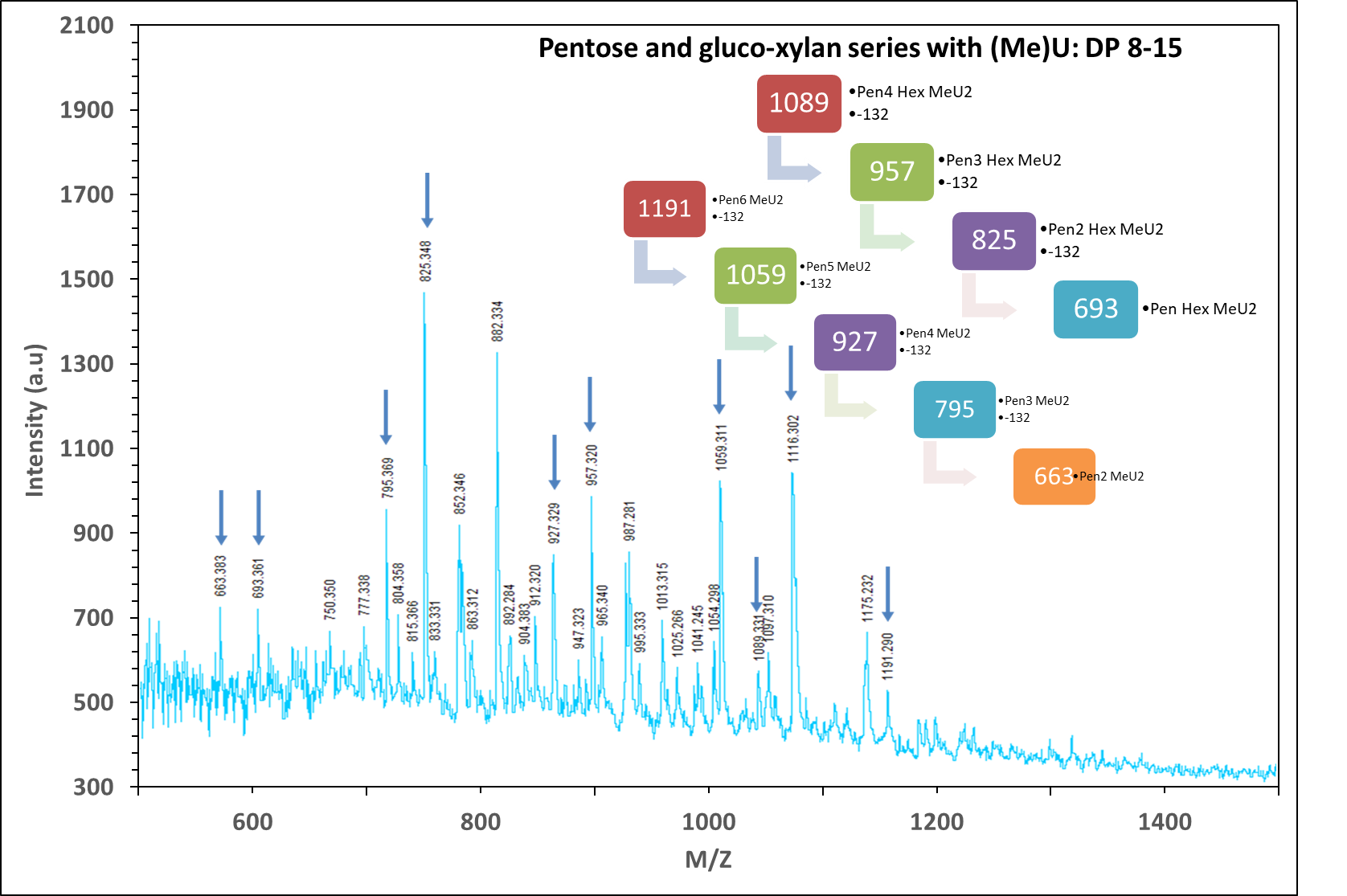


**Figure S3**: MALDI Analyses of FA-C confirmed Pentose and gluco-xylan series with (Me)U: DP 8-15.


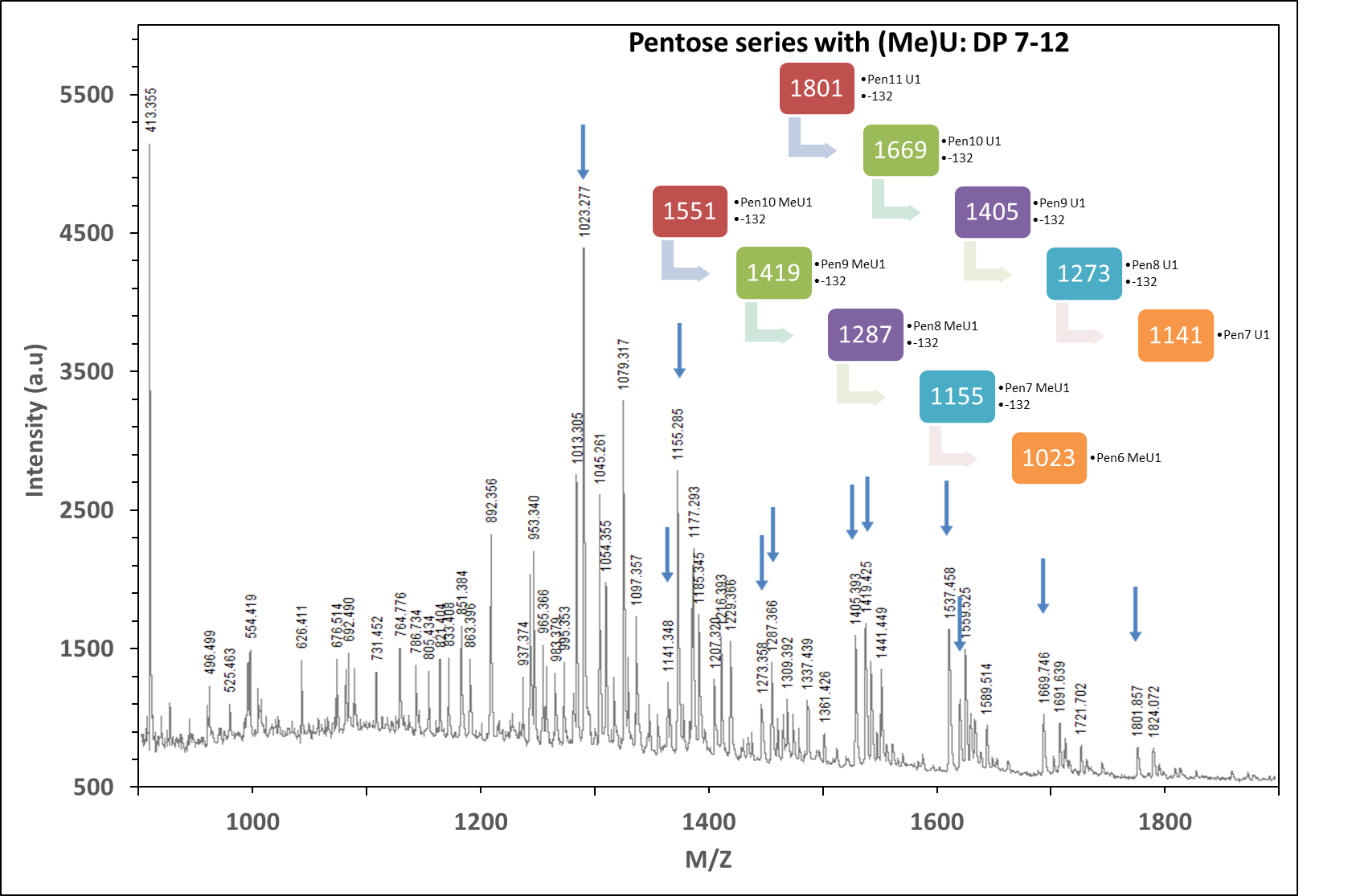


**Figure S4**. MALDI Analyses of FA-D confirmed Pentose series with (Me)U: DP 7-12.


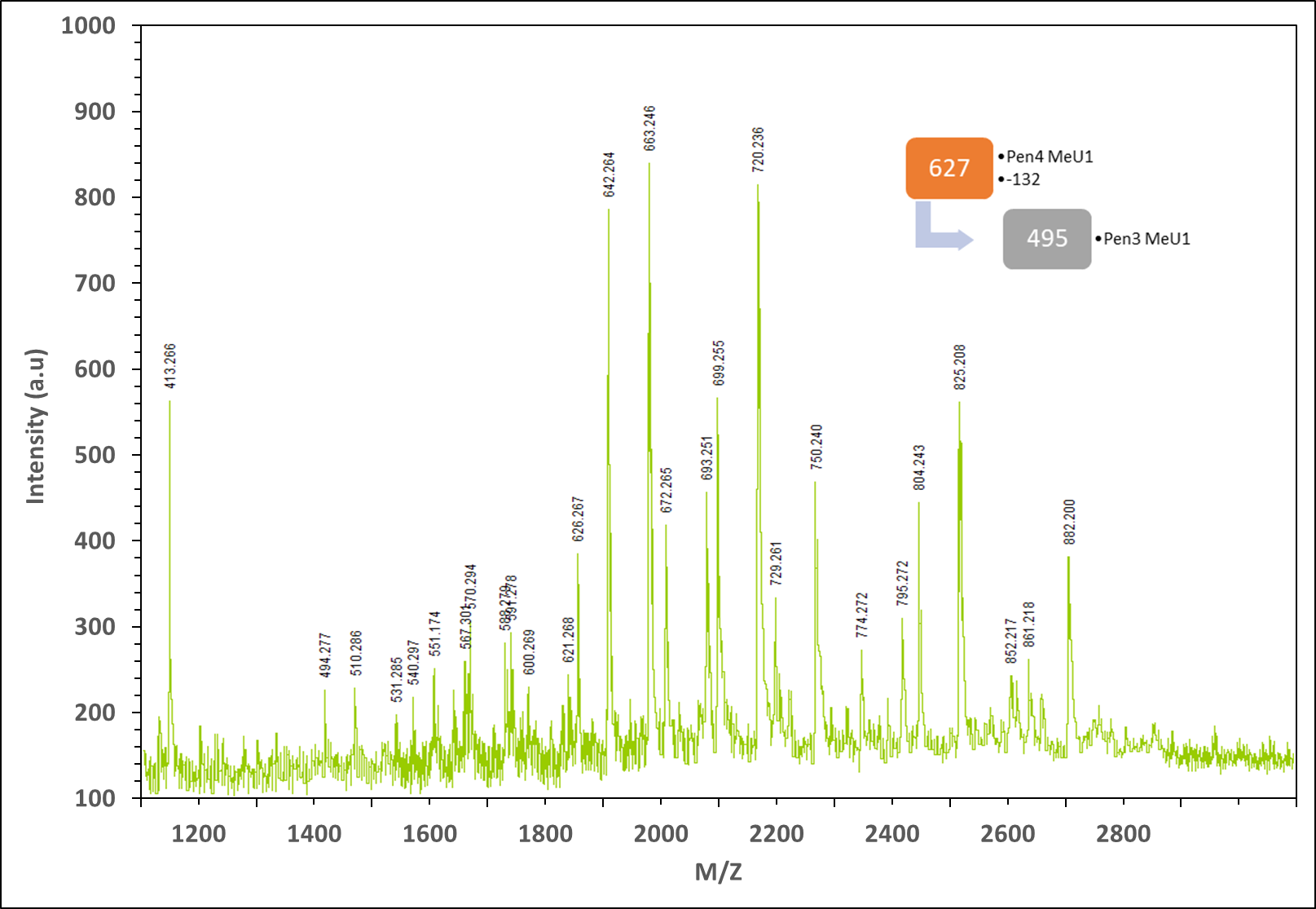


**Figure S5.** MALDI Analyses of H-2 confirmed Pentose series with MeU: DP 3-4, and Xyloglucan or gluco-xylan with substitution: DP 4-6.


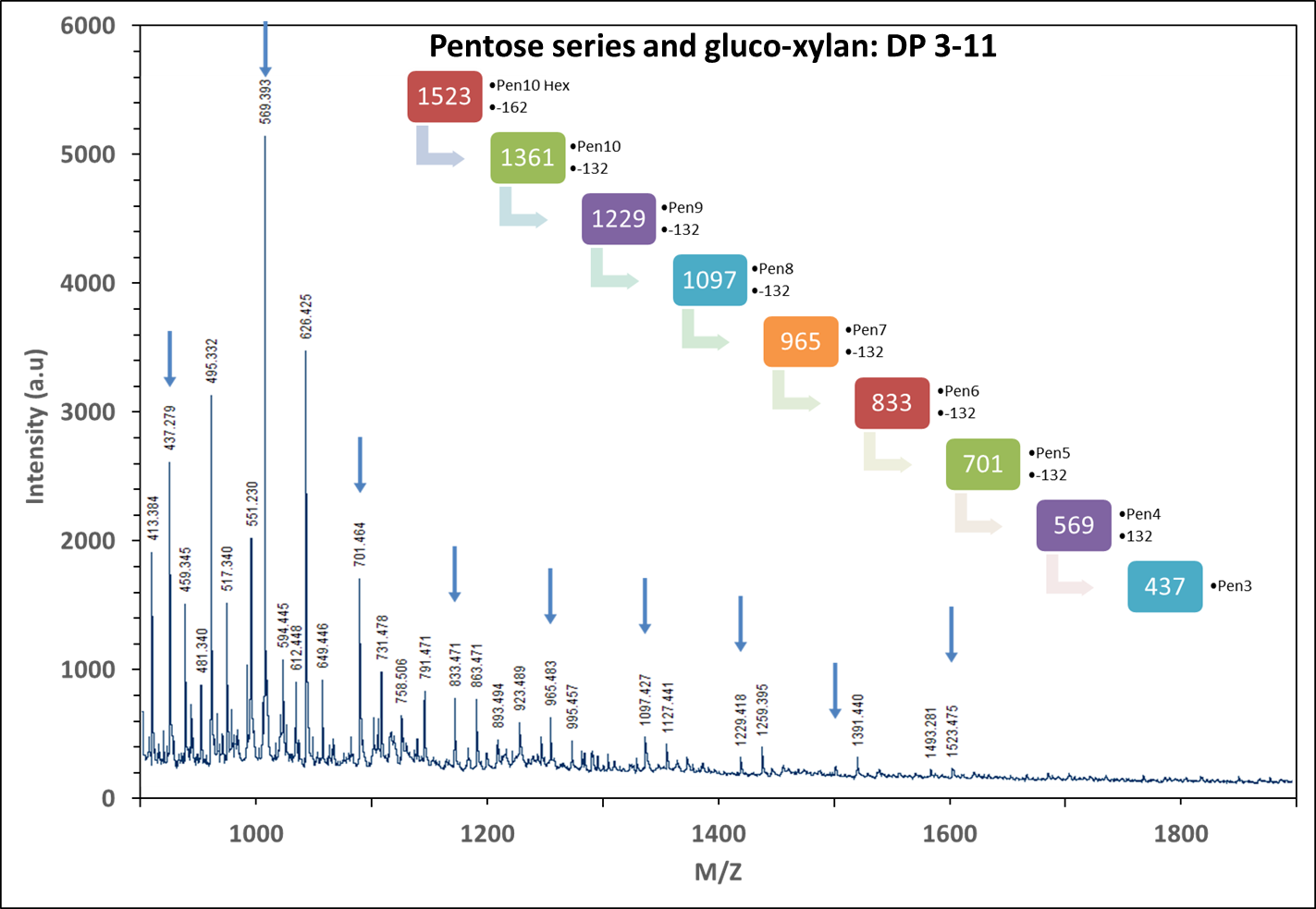


**Figure S6**. MALDI Analyses of H-3 confirmed Pentose series and gluco-xylan: DP 3-11.


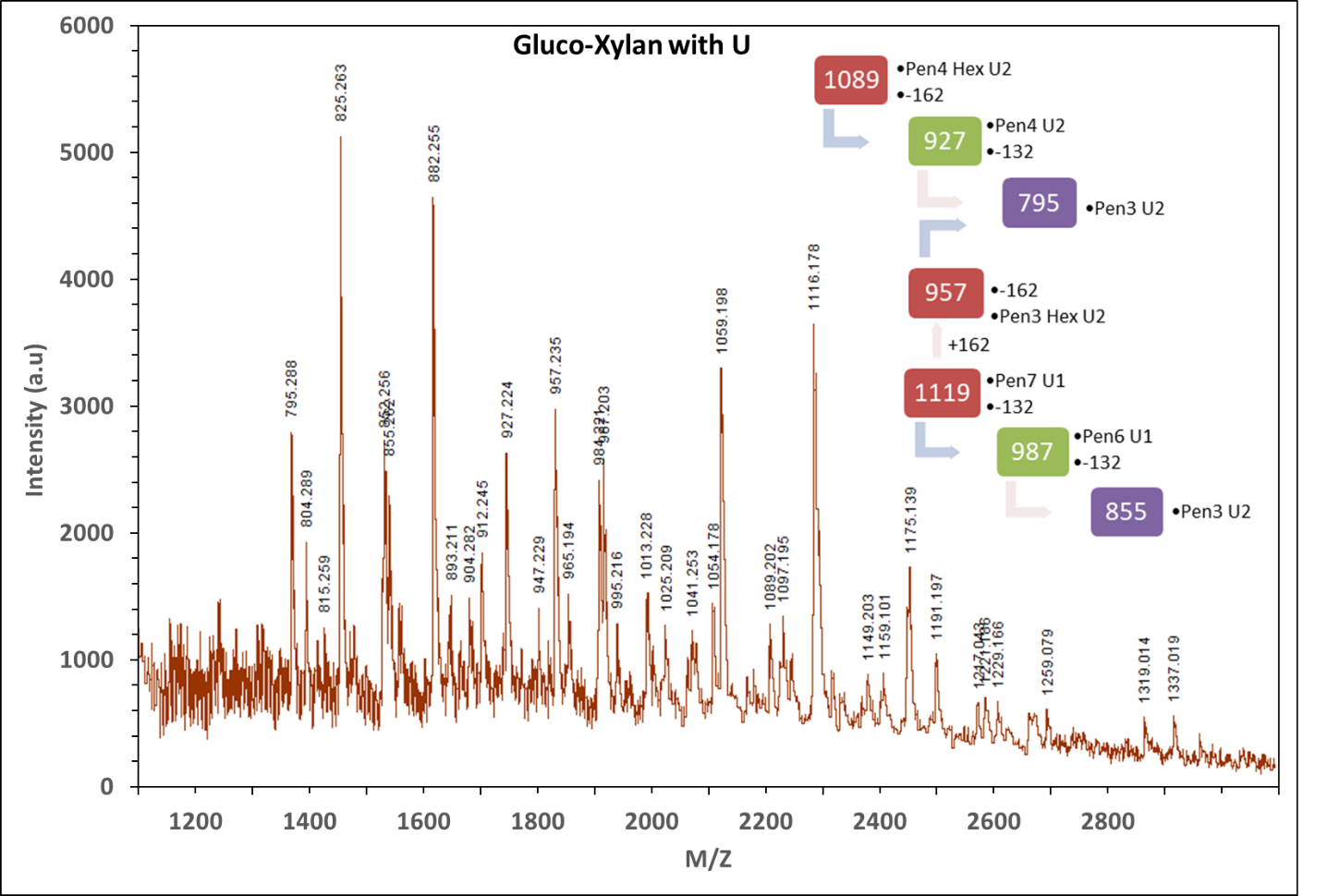


**Figure S7**. MALDI Analyses of H-4 confirmed Gluco-Xylan with U


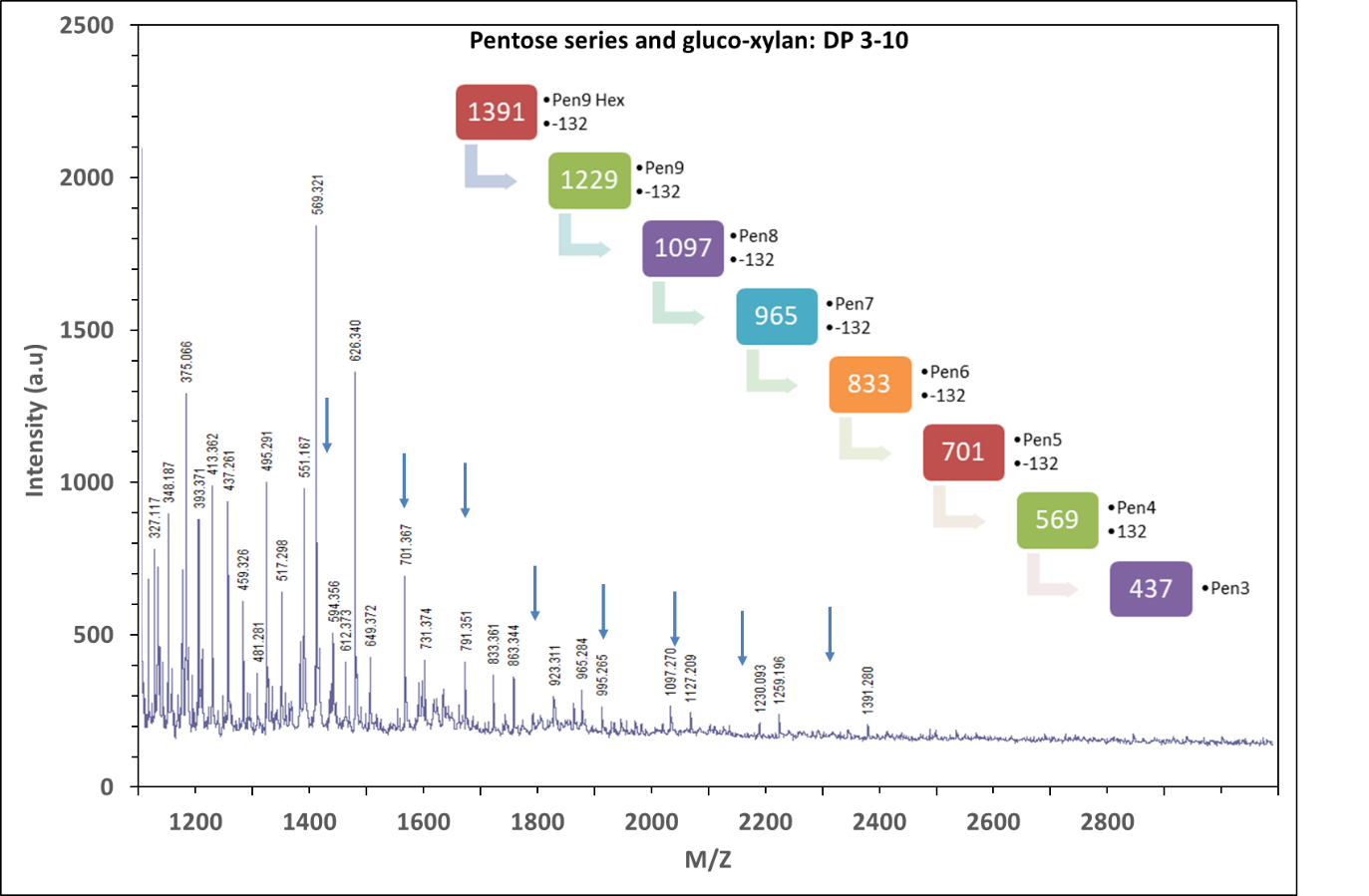


**Figure S8**. MALDI Analyses of H-6 confirmed Pentose series and gluco-xylan: DP 3-10

**B**

**A**

**Figure S9**: HPLC-MS analysis of soluble recalcitrant oligosaccharides linkages, hexose and –OAc fragmentation are repetitively observed. Here, A, Mass Spectrum of ACN-B fraction; B, Mass Spectrum of ACN-B low range fraction

**Methods:**

**GC-MS Control Parameters for TMS sugar composition analysis**

Sample Inlet: GC

Injection Source: GC ALS

Mass Spectrometer: Enabled

**Oven**

Equilibration Time: 0.5 min

Run Time: 43.667 min

1 min (Post Run): 270 ^o^C

Front Injector and Front SS Inlet, Carrier gas: He

Mode: Split

Heater: On, 250 ^o^C

Pressure: On, 9.3825 psi

Total Flow: On, 54 mL/min

Septum Purge Flow: On, 3 mL/min

Gas Saver: On, 20 mL/min After 4 min

Split Ratio: 50 :1

Split Flow: 50 mL/min

**Column**

EC™-1 Capillary Columns, dimethylpolysiloxane, 30 m x 250 µm x 0.25 µm; 360 ^o^C

**MS ACQUISITION PARAMETERS**

**General Information**

Acquistion Mode: Scan

Solvent Delay: 5.00 min

EMV Mode: Relative

Relative Voltage: 0

Resulting EM Voltage: 1471

**Scan Parameters**

Low Mass: 50.0

High Mass: 550.0

Threshold: 150

[MSZones]

MS Source: 230 ^o^C (maximum 250 ^o^C)

MS Quad: 150 ^o^C (maximum 200 ^o^C)

**HPLC-MS method for oligosaccharides analysis:**

**HPLC control parameters**

Run Time: 20.00 min

Comment: BEH Amide column, 2.1 mm x 100 mm

Column Temperature: 40.0 ± 5.0^o^C

Solvent A: 10 mM aqueous NH_4_OAc

Solvent B: Acetonitrile

**Gradient Table**

Time(min), Flow Rate, %A, %B

1. Initial, 0.2, 5.0, 95.0

2. 1.00, 0.2, 5.0, 95.0

3. 14.00, 0.2, 65.0, 35.0

4. 16.00, 0.2, 65.0, 35.0

5. 16.01, 0.2, 5.0 95.0

6. 20.00, 0.2, 5.0, 95.0

Syringe Size = 100μl

**MS acquisition parameters**

Function: Met ID, Time 0.00 to 20.00, Mass 100.00 to 3500.00 ES­

Scan Time (sec): 0.500

Interscan Time (sec): 0.014

Start Mass: 100.0

End Mass: 3500.0

Start Time (mins): 0.00

End Time (mins): 20.00

Data Format: Centroid

ADC Pusher Frequency (µs): 75.0

ADC Pusher Width (µs): 4.00

Low Energy mass range: 100.000000 - 3500.000000

High energy mass range: 100.000000 - 3500.000000

Use Tune Page Cone Voltage: Yes

Use Tune Page Low CE: Yes

Use Tune Page High CE: No

Trap CE Ramp Initial Voltage: 10.0

Trap CE Ramp Final Voltage: 80.0

Sensitivity: Normal

Dynamic Range: Normal

**Lock Mass**

Mass Window +/-: 0.5

Scans to Average: 3.0
